# Gestational diabetes mellitus in pregnancies conceived after infertility treatment: a population-based study in the United States, 2015–2020

**DOI:** 10.1016/j.xfre.2023.11.008

**Published:** 2023-11-17

**Authors:** Devika Sachdev, Mark V. Sauer, Cande V. Ananth

**Affiliations:** a Department of Obstetrics, Gynecology, and Reproductive Sciences, Rutgers Robert Wood Johnson Medical School, New Brunswick, New Jersey; b Division of Epidemiology and Biostatistics, Department of Obstetrics, Gynecology, and Reproductive Sciences, Rutgers Robert Wood Johnson Medical School, New Brunswick, New Jersey; c Cardiovascular Institute of New Jersey, Rutgers Robert Wood Johnson Medical School, New Brunswick, New Jersey; d Department of Biostatistics and Epidemiology, Rutgers School of Public Health, Piscataway, New Jersey; e Environmental and Occupational Health Sciences Institute, Rutgers Robert Wood Johnson Medical School, Piscataway, New Jersey

**Keywords:** Infertility, assisted reproductive technology, fertility-enhancing drugs, gestational diabetes mellitus, BMI

## Abstract

**Objective:**

To evaluate the risk of gestational diabetes mellitus (GDM) in singleton pregnancies conceived using infertility treatment and examine the influence of race and ethnicity as well as prepregnancy body mass index (BMI).

**Design:**

Cross-sectional study using the US vital records data of women that delivered singleton births.

**Setting:**

United States, 2015–2020.

**Interventions:**

Any infertility treatment was divided into two groups: those that used fertility-enhancing drugs, artificial insemination, or intrauterine insemination, and those that used assisted reproductive technology (ART).

**Main Outcome Measures(s):**

Gestational diabetes mellitus, defined as a diagnosis of diabetes mellitus during pregnancy, includes both diet-controlled GDM and medication-controlled GDM in singleton pregnancies conceived with infertility treatment or spontaneously and delivered between 20- and 44-weeks’ gestation. We also examined whether the infertility treatment-GDM association was modified by maternal race and ethnicity as well as prepregnancy BMI. Associations were expressed as a rate ratio (RR) and 95% confidence interval (CI), derived from log-linear models after adjustment for potential confounders.

**Results:**

A total of 21,943,384 singleton births were included, with 1.5% (n = 318,086) undergoing infertility treatment. Rates of GDM among women undergoing infertility treatment and those who conceived spontaneously were 11.0% (n = 34,946) and 6.5% (n = 1,398,613), respectively (adjusted RR 1.24, 95% CI 1.23, 1.26). The RRs were adjusted for maternal age, parity, education, race and ethnicity, smoking, BMI, chronic hypertension, and year of delivery. The risk of GDM was modestly increased for those using fertility-enhancing drugs (adjusted RR 1.28, 95% CI 1.27, 1.30) compared with ART (adjusted RR 1.18, 95% CI 1.17, 1.20), and this risk was especially apparent for non-Hispanic White women (adjusted RR 1.29, 95% CI 1.26, 1.31) and Hispanic women (adjusted RR 1.35, 95% CI 1.29, 1.41). The number of women who needed to be exposed to infertility treatment to diagnose one case of GDM was 46. Prepregnancy BMI did not modify the infertility treatment-GDM association overall and within strata of race and ethnicity. These general patterns were stronger after potential corrections for misclassification of infertility treatment and unmeasured confounding.

**Conclusions:**

Infertility treatment, among those who received fertility-enhancing drugs, is associated with an increased GDM risk. The persistently higher risk of GDM among women who seek infertility treatment, irrespective of prepregnancy weight classification, deserves attention. Infertility specialists must be vigilant with preconception counseling and ensure that all patients, regardless of race and ethnicity or BMI, are adequately tested for GDM early in pregnancy using a fasting blood glucose level or a traditional 50-g oral glucose tolerance test. Testing may be completed by the infertility specialist or deferred to the primary prenatal care provider at the first prenatal visit.

Because infertility treatment has steadily increased over recent years (1), the evidence linking adverse pregnancy outcomes with this mode of conception has also grown. The proportion of pregnancies being affected by gestational diabetes mellitus (GDM) has vastly increased across all racial groups over the past decade, given the trend in obesity rates, increased sedentary lifestyle, and older maternal age (2). It is estimated that approximately 7% of pregnancies are associated with diabetes in general, with a very large proportion of these attributable to gestational diabetes (3). Gestational diabetes mellitus, especially when uncontrolled, is associated with several poor obstetric and neonatal outcomes, including preeclampsia, cesarean section, macrosomia, birth trauma, stillbirth, and an increased risk of developing type 2 diabetes later in life (3). What is less known and less studied is the contribution of various infertility treatments to the development of GDM.

Assisted reproductive technology (ART) is an umbrella term for a variety of treatments, protocols, laboratory techniques, and medications used to treat infertility. These include in vitro fertilization, intracytoplasmic sperm injection (ICSI), preimplantation genetic testing, embryo cryopreservation, embryo donation, and the use of a gestational carrier (4). Pregnancies resulting from infertility treatment have steadily increased in the past decade, given the vast advances in the field and improved access to infertility treatments in general. According to the United States Centers for Disease Control and Prevention’s (CDC) 2019 Fertility Clinic and National Summary Report, the use of ART has more than doubled in the past decade (1). The number of ART cycles resulting in live births was approximately 1.7 times greater in 2019 than in 2010, and approximately 2% of all live births in the United States in 2019 involved ART (1). With the increasing use of ART, clinicians must be cognizant of the pregnancy risks that are associated with it.

Although various perinatal complications, as well as increased risks of chronic diseases in the short- and long-term, have been associated with ART pregnancies, the risk of GDM following ART pregnancies has not been well-studied. Certain risk factors in women undergoing ART treatment may predispose them to the development of GDM, including hormone use, a history of ovarian hyperstimulation syndrome, and polycystic ovary syndrome (PCOS) (5). However, some studies have shown that the use of ART may be an independent risk factor for the development of GDM (6, 7). Not surprisingly, prepregnancy body mass index (BMI) has been a focus of many investigations, with data mainly revolving around those who are overweight or obese, because it is presumed that those with higher BMIs are more likely to develop GDM (8, 9).

Overall, there have been noted differences in rates of GDM when stratified by race (3). In a retrospective cohort study over a 4-year time period, Caughey et al. (10) found that the rate of GDM was greatest among Asian women. In addition, after controlling for confounders, the rate of GDM was greater among Asian and Hispanic women compared with White women (10). The rate of GDM has been increasing over the past decade across all racial subgroups (2). However, few studies have examined the association between race and GDM outcomes in pregnancies using ARTs.

We hypothesized that treatment for infertility will be associated with increased GDM risk, and the risks may vary across prepregnancy BMI and the patient’s race and ethnicity. Because the prevalence of many obstetric complications vary by race, we believed stratifying our analysis using race and ethnicity would be informative. We tested this hypothesis in a large cohort of women who delivered a singleton pregnancy in the US.

## Materials and methods

The United States Centers for Disease Control and Prevention’s National Vital Statistics System (NVSS) data were used to extrapolate data for this cross-sectional study from the years 2015–2020. The US Standard Certificate Live Birth Form (revised in 2003) is used by all states and sent to the NVSS for data assembly. The US Standard Certificate Live Birth and Fetal Death forms report basic patient demographics, newborn outcomes, and a variety of medical and health information, including the presence of pregestational or gestational diabetes and use of infertility treatments before pregnancy (11, 12).

### Inclusion Criteria

We restricted the study to women that delivered a singleton pregnancy (with a live birth or stillbirth) at 20–44 weeks’ gestation. No patient identifiers are used in the reporting of data to the NVSS, and as such, Institutional Review Board approval was not required.

### Exclusion Criteria

Women diagnosed with diabetes before pregnancy and those who delivered before 20 weeks or after 44 weeks’ gestation were excluded. We further excluded twin or higher-order pregnancies because such pregnancies carry different risk profiles for infertility treatment and GDM risks.

### Exposure

The exposure group in this study was pregnancies achieved after infertility treatment. Infertility treatment was examined as those that used fertility-enhancing drugs, artificial insemination, or intrauterine insemination (IUI); and those that used ART, which included in vitro fertilization (IVF) and gamete intrafallopian transfer (GIFT). The data reported do not distinguish between IVF and GIFT. Fertility-enhancing drugs include clomiphene citrate, letrozole, injectable gonadotropins, or a combination of these. Data on infertility treatment are derived from three (sequential) sources: prenatal care records, labor and delivery nursing admission triage notes, or admission history.

### Outcomes

The primary outcome studied was GDM, defined as a diagnosis of diabetes mellitus during the pregnancy, including both diet-controlled GDM and medication-controlled GDM; the data reported to the NVSS does not distinguish between the two. Validation of GDM on the vital statistics data is moderate for sensitivity (65%) and very high for specificity (98%) (13). Modifications using prepregnancy BMI and maternal race and ethnicity were explored.

### Statistical Analysis

The risk of GDM in individuals who received any type of infertility treatment, as well as the risk in the two subgroups, was derived from log-linear Poisson regression models with robust variance in comparison to the risk in pregnancies conceived spontaneously. The associations were expressed as risk ratios (RRs) and 95% confidence intervals (CIs). The RRs were adjusted for maternal age, parity, education, race and ethnicity, smoking, BMI, chronic hypertension, and year of delivery. The analysis was then separated using maternal race and ethnicity and within strata of prepregnancy BMI. We also estimated the adjusted rate difference and 95% CI as a measure to determine the excess number of GDM cases between the IVF and spontaneous conception groups.

### Missing Data

Owing to missing data in covariates, we undertook a multiple imputation analysis. This was based on creating 25 data sets with imputed missing data on the basis of fully conditional specification and subsequently combined on the basis of Rubin’s method. The imputation models included infertility treatment, GDM, as well as other covariates, and the models were fit based on the expectation-maximization algorithm (14).

We also estimated the number needed to be exposed statistic (15). The number needed to be exposed (NNE) is interpreted as the number of IVF conceptions that result in one case of GDM. This measure is helpful to gauge the population impact of IVF treatment on the risk of GDM.

### Sensitivity Analysis

Data on infertility treatment are underreported in the vital statistics data (sensitivity 36.5% and specificity >98%) (16) compared with the data reported in the Society for Assisted Reproductive Technology Clinic Outcome Reporting System database. In addition, we also examined whether bias because of unmeasured confounding may have impacted the associations between infertility treatment and GDM. To examine the extent to which misclassification and unmeasured confounding biases may have affected the effect measures, we undertook a probabilistic bias analysis (17, 18).

The probabilistic bias analysis was designed to simultaneously correct for exposure misclassification and unmeasured confounding biases. For the exposure misclassification, the simulations were based on ranging the sensitivity and specificity estimates for IVF of 0.3–0.9 and 0.95–1.00, respectively, under a uniform distribution. For unmeasured confounding, we assumed that the prevalence estimates of the unmeasured confounder(s) under a logit-normal model among the IVF exposed and unexposed groups would be similar (−0.75, 0.80, 0.00, 1.00), and the risks would range from 0.10–2.00 under a uniform distribution. We generated these distributions 100,000 times each and reported the median bias-corrected RR (RR_bC_) and 95% simulation interval.

The statistical analyses were implemented in SAS (version 9.4; SAS Institute, Cary, NC) between October 2022 and March 2023. The probabilistic bias analysis was accomplished using the Episensr package in R Studio (19).

## Results

A total of 21,943,384 singleton live births were included in the analysis with 1.5% (n = 318,086) undergoing infertility treatment (Supplemental Fig. 1, available online). The use of infertility treatment is higher in those who are primiparous, women of advanced maternal age, non-Hispanic Whites, and normal prepregnancy BMI (Table 1).

**Table 1:** Distribution of maternal characteristics in relation to infertility status among singleton births: United States, 2015–2020.

Maternal characteristics	Spontaneously conceived pregnancies: number (%)	Infertility treatment: number (%)		
		Any treatment	Assisted reproductive technology	Fertility-enhancing drugs
**Total pregnancies**	21,625,298 (98.6)	318,086 (1.5)	188,380 (0.9)	132,954 (0.6)
**Year of delivery**				
2015	3,712,003 (17.2)	41,633 (13.1)	21,225 (11.2)	19,709 (14.8)
2016	3,738,837 (17.3)	47,253 (14.9)	25,646 (13.6)	21,411 (16.1)
2017	3,645,420 (16.9)	52,413 (16.5)	29,991 (15.9)	22,793 (17.1)
2018	3,584,143 (16.6)	56,184 (17.7)	33,353 (17.7)	23,528 (17.7)
2019	3,538,415 (16.4)	60,078 (18.9)	38,455 (20.4)	23,000 (17.3)
2020	3,406,480 (15.8)	60,525 (19.0)	39,710 (21.1)	22,513 (16.9)
Missing	0	0	21,407	21,407
**Maternal age (y)**				
<15	11,749 (0.1)	1 (<0.1)	0 (0.0)	0 (0.00)
15–19	1,119,266 (5.2)	231 (0.1)	44 (<0.1)	110 (0.1)
20–24	4,372,210 (20.2)	7,184 (2.3)	1,548 (0.8)	4,814 (3.6)
25–29	6,324,710 (29.2)	44,550 (14.0)	16,617 (8.8)	26,333 (19.8)
30–34	6,119,821 (28.3)	110,261 (34.7)	60,050 (31.9)	50,651 (38.1)
35–39	3,033,444 (14.0)	102,216 (32.1)	67,547 (35.9)	37,059 (27.9)
40–44	609,353 (2.8)	41,320 (13.0)	31,984 (17.0)	11,395 (8.6)
45–49	32,821 (0.2)	10,131 (3.2)	8,672 (4.6)	2,127 (1.6)
≥50	1,924 (<0.1)	2,192 (0.7)	1,918 (1.0)	465 (0.3)
Missing	0	0	21,407	21.407
**Parity (live-born)**				
Parity 1	8,306,373 (38.4)	192,799 (60.6)	112,986 (60.0)	82,570 (62.1)
Parity 2	6,881,674 (31.8)	86,906 (27.3)	51,357 (27.3)	36,032 (27.1)
Parity ≥3	6,437,251 (29.8)	38,381 (12.1)	24,037 (12.8)	14,352 (10.8)
Missing	0	0	21,407	21,407
**Maternal education**				
Below high school	712,210 (3.3)	1,113 (0.3)	557 (0.3)	451 (0.3)
High school	7,702,973 (35.6)	23,901 (7.5)	11,355 (6.0)	11,525 (8.7)
College	10,431,541 (48.2)	173,331 (54.5)	97,350 (51.7)	76,791 (57.8)
Beyond college	2,496,739 (11.5)	112,728 (35.4)	73,145 (38.8)	42,203 (31.7)
Missing	281,835	7,013	310,086	310,086
**Maternal race/ethnicity**				
Non-Hispanic White	11,087,904 (51.3)	228,271 (71.8)	129,667 (68.8)	100,166 (75.3)
Non-Hispanic Black	3,109,235 (14.4)	14,793 (4.7)	8,884 (4.7)	5,722 (4.3)
Hispanic	5,186,144 (24.0)	26,710 (8.4)	15,540 (8.2)	11,423 (8.6)
Others	2,223,009 (10.3)	47,921 (15.1)	34,047 (18.1)	15,493 (11.7)
Missing	19,006	391	40,786	40,786
**Smoking status**				
Nonsmoker	19,572,254 (90.5)	311,296 (97.9)	185,382 (98.4)	129,504 (97.4)
Before pregnancy only	454,332 (2.1)	2,673 (0.8)	1,125 (0.6)	1,456 (1.1)
Before and during pregnancy	1,477,128 (6.8)	3,228 (1.0)	1,336 (0.7)	1,655 (1.2)
Missing	121,584	889	143,809	143,809
**Marital status**				
Married	11,760,969 (54.4)	269,174 (84.6)	154,189 (81.8)	116,506 (87.6)
Single	8,094,182 (34.4)	23,206 (7.3)	13,180 (7.0)	9,497 (7.1)
Missing	1,770,147	25,706	1,817,191	1,817,191
**Prepregnancy body mass index (kg/m**^**2**^**)**				
Underweight (<18.5)	702,988 (3.3)	6,878 (2.2)	4,359 (2.3)	2,548 (1.9)
Normal weight (18.5–24.9)	9,064,418 (41.9)	148,663 (46.7)	103,574 (55.0)	63,238 (47.6)
Overweight (25.0–29.9)	5,591,627 (25.9)	80,399 (25.3)	45,291 (24.0)	31,814 (23.9)
Class I obesity (30.0–34.9)	3,131,948 (14.5)	42,125 (13.2)	21,000 (11.1)	17,968 (13.5)
Class II and III obesity (≥35.0)	2,598,370 (12.0)	34,932 (11.0)	14,156 (7.5)	17,386 (13.1)
Missing	535,947	5,089	21,407	21,407
**Chronic hypertension**				
Present	393,342 (1.8)	9,955 (3.1)	5,779 (3.1)	4,234 (3.2)
Absent	21,231,956 (98.2)	308,131 (96.9)	182,601 (96.8)	128,720 (96.8)
Missing	0	0	21,407	21,407

The rates of GDM among women who underwent any infertility treatment and spontaneous conceptions were 11.0% and 6.5%, respectively (Table 2). Rates of GDM were slightly higher among women who received fertility-enhancing drugs (11.0% for fertility-enhancing drugs compared with 10.9% for ART). These rates were also higher among Hispanic women than non-Hispanic White or non-Hispanic Black women. After adjustments for confounders, there were an excess of 3.8% of GDM cases among women with any infertility treatment compared with spontaneous conceptions, with the excess risk being higher among non-Hispanic Black and Hispanic women (Table 3). Any infertility treatment was associated with a 24% increased risk of GDM (RR 1.24, 95% CI 1.23, 1.26), but corrections for exposure misclassification and unmeasured confounding resulted in stronger associations (RR_bc_ 1.62, 95% simulation interval 1.21, 2.17). After corrections for biases, the association between infertility treatment and GDM risk was strongest for non-Hispanic Black women (Table 3).

**Table 2:** Rates of gestational diabetes mellitus by infertility status overall and by race and ethnicity: United States, 2015–2020.

Race/ethnicity	Total pregnancies number (%_row_)^a^	Gestational diabetes mellitus	
		Absent number (%_row_)	Present number (%_row_)
**All race/ethnicities**			
Spontaneous conceptions	21,625,298 (6.4)	20,226,685 (93.5)	1,398,613 (6.5)
Any infertility treatment	318,086 (0.2)	283,140 (89.0)	34,946 (11.0)
Assisted reproductive technology	188,380 (0.1)	167,803 (89.1)	20,577 (10.9)
Fertility-enhancing drugs	132,954 (0.1)	118,330 (89.0)	14,624 (11.0)
**Non-Hispanic White**			
Spontaneous conceptions	11,087,904 (5.6)	10,449,886 (94.2)	638,018 (5.8)
Any infertility treatment	222,271 (0.2)	206,798 (90.6)	21,473 (9.4)
Assisted reproductive technology	129,667 (0.1)	117,853 (90.9)	11,814 (9.1)
Fertility-enhancing drugs	100,166 (0.1)	90,488 (90.3)	9678 (9.7)
**Non-Hispanic Black**			
Spontaneous conceptions	3,109,235 (5.2)	2,945,429 (94.7)	163,806 (5.3)
Any infertility treatment	14,793 (0.05)	13,152 (88.9)	1641 (11.1)
Assisted reproductive technology	8884 (0.03)	7865 (88.5)	1019 (11.5)
Fertility-enhancing drugs	5722 (0.02)	5079 (88.8)	643 (11.2)
**Hispanic**			
Spontaneous conceptions	5,186,144 (7.1)	4,817,953 (92.9)	368,191 (7.1)
Any infertility treatment	26,710 (0.07)	23,302 (87.2)	3408 (12.8)
Assisted reproductive technology	15,540 (0.04)	13,610 (87.6)	1930 (12.4)
Fertility-enhancing drugs	11,424 (0.03)	9917 (86.8)	1506 (13.2)

**Table 3:** Risk of gestational diabetes mellitus by infertility status and by race and ethnicity among singleton births: United States, 2015–2020.

	Adjusted rate difference (95% CI)	Adjusted NNE (95% CI)	Adjusted rate ratio (95% CI)^a^^,^^b^	Bias-corrected rate ratio (95% SI)^c^
**Spontaneous conceptions**	0.0 (Reference)	0.0 (Reference)	1.00 (Reference)	1.00 (Reference)
**All race/ethnicities**				
Any infertility treatment	3.8 (3.7, 3.9)	46 (44, 48)	1.24 (1.23, 1.26)	1.62 (1.21, 2.17)
Assisted reproductive technology	3.9 (3.8, 4.0)	79 (72, 89)	1.18 (1.17, 1.20)	1.70 (1.27, 2.27)
Fertility-enhancing drugs	3.5 (3.3, 3.6)	31 (30, 33)	1.28 (1.27, 1.30)	1.83 (1.37, 2.44)
**Non-Hispanic White**				
Any infertility treatment	3.0 (2.9, 3.1)	47 (44, 50)	1.26 (1.24, 1.27)	1.49 (1.12, 1.99)
Assisted reproductive technology	3.1 (3.0, 3.2)	77 (68, 88)	1.20 (1.18, 1.23)	1.45 (1.09, 1.94)
Fertility-enhancing drugs	2.7 (2.6, 2.9)	35 (32, 37)	1.29 (1.26, 1.31)	1.58 (1.18, 2.11)
**Non-Hispanic Black**				
Any infertility treatment	5.2 (4.7, 5.7)	102 (72, 171)	1.25 (1.20, 1.31)	2.00 (1.49, 2.67)
Assisted reproductive technology	5.6 (4.9, 6.3)	190 (99, 2431)	1.22 (1.18, 1.23)	2.17 (1.62, 2.91)
Fertility-enhancing drugs	4.9 (4.1, 5.7)	51 (38, 80)	1.30 (1.21, 1.40)	2.29 (1.70, 3.07)
**Hispanic**				
Any infertility treatment	5.0 (4.7, 5.4)	59 (48, 75)	1.26 (1.22, 1.30)	1.61 (1.20, 2.15)
Assisted reproductive technology	5.0 (4.6, 5.5)	196 (105, 1439)	1.18 (1.14, 1.24)	1.56 (1.17, 2.09)
Fertility-enhancing drugs	5.1 (4.5, 5.6)	31 (26, 38)	1.35 (1.29, 1.41)	1.66 (1.24, 2.23)

CI = confidence interval; NNE = number needed to be exposed; SI = simulation interval.

a Rate ratios are adjusted for maternal age, live-born parity, education, race and ethnicity, body mass index, chronic hypertension, and year of delivery

b Confounder-adjusted rate difference and rate ratios are based on imputation analysis for missing covariates (shown in Table 1)

c Bias-corrected RRs refers to multiple probabilistic bias-corrected risk ratio, after simultaneous corrections for nondifferential exposure misclassification (infertility treatment) and unmeasured confounding biases

The associations between infertility treatment and GDM within strata of prepregnancy BMI as well as race and ethnicity are presented in Table 4. The excess GDM rate, in general, increased with higher BMI for all races and ethnicities. Correspondingly, the adjusted NNE decreased with a higher BMI. For instance, among non-Hispanic White women, the NNE for underweight women was 151, suggesting that for every woman to be diagnosed with GDM, 151 women need to undergo infertility treatment. In contrast, the NNE among the classes II and III obesity groups (BMI ≥35 kg/m^2^) was much smaller (NNE 29). These general patterns of associations were similar for women who underwent ART and fertility-enhancing drugs (Supplemental Tables 1 and 2, respectively, available online). For those with a normal BMI or above (≥18.5 kg/m^2^), the risk of GDM was similarly increased across all racial groups for any type of infertility treatment in general. For underweight women (BMI <18.5 kg/m^2^), non-Hispanic White women are still at an increased risk compared with spontaneously conceived pregnancies for any infertility treatment type (adjusted RR 1.26, 95% 1.10, 1.44), but this risk was not seen with underweight non-Hispanic Black or Hispanic women.

**Table 4:** Risk of gestational diabetes mellitus by any infertility treatment and stratified by race and ethnicity as well as prepregnancy body mass index among singleton births: United States, 2015–2020.

Any infertility treatment and prepregnancy body mass index (kg/m^2^)	Adjusted rate difference (95% CI)	Adjusted NNE (95% CI)	Adjusted rate ratio (95% CI)^a^^,^^b^	Bias-corrected rate ratio (95% SI)^c^
**Spontaneous conceptions**	0.0 (Reference)	0 (Reference)	1.00 (Reference)	1.00 (Reference)
**Non-Hispanic White**				
Underweight (<18.5)	1.1 (0.5, 1.7)	151 (87, 554)	1.26 (1.10, 1.44)	1.71 (1.26, 2.32)
Normal weight (18.5–24.9)	1.1 (1.0, 1.2)	108 (95, 124.)	1.27 (1.23, 1.30)	1.51 (1.13, 2.02)
Overweight (25–29.9)	2.3 (2.0, 2.5)	52 (46, 60)	1.29 (1.26, 1.33)	1.61 (1.21, 2.16)
Class I obesity (30–34.9)	3.0 (2.6, 3.4)	42 (36, 50)	1.23 (1.19, 1.26)	1.56 (1.17, 2.09)
Class II and III obesity (≥35)	4.6 (4.1, 5.0)	29 (26, 34)	1.24 (1.20, 1.28)	1.55 (1.16, 2.07)
**Non-Hispanic Black**				
Underweight (<18.5)	0.7 (−2.1, 3.6)	642 (48, −56)^d^	1.03 (0.46, 2.34)	2.27 (1.10, 4.68)
Normal weight (18.5–24.9)	1.4 (0.8, 2.1)	240 (113, −1866)	1.28 (1.14, 1.44)	2.35 (1.74, 3.18)
Overweight (25–29.9)	2.7 (1.8, 3.6)	108 (64, 365)	1.28 (1.18, 1.40)	2.06 (1.53, 2.77)
Class I obesity (30–34.9)	2.9 (1.6, 4.1)	82 (45, 434)	1.18 (1.07, 1.29)	1.91 (1.41, 2.57)
Class II and III obesity (≥35)	4.3 (2.7, 5.8)	46 (29, 112)	1.23 (1.13, 1.35)	1.82 (1.34, 2.45)
**Hispanic**				
Underweight (<18.5)	1.7 (−0.7, 4.2)	101 (39, −170)	1.35 (0.89, 2.04)	2.92 (1.87, 4.56)
Normal weight (18.5–24.9)	0.9 (0.4, 1.4)	184 (108, 622)	1.22 (1.14, 1.31)	1.80 (1.34, 2.42)
Overweight (25–29.9)	2.9 (2.1, 3.6)	58 (43, 94)	1.32 (1.25, 1.40)	1.80 (1.35, 2.41)
Class I obesity (30–34.9)	4.2 (3.0, 5.4)	28 (22, 40)	1.30 (1.22, 1.38)	1.92 (1.44, 2.59)
Class II and III obesity (≥35)	3.5 (2.0, 4.9)	54 (32, 182)	1.15 (1.07, 1.23)	1.53 (1.14, 2.05)

CI = confidence interval; NNE = number needed to be exposed; SI = simulation interval.

a Rate ratios are adjusted for maternal age, live-born parity, education, race and ethnicity, body mass index, chronic hypertension, and year of delivery

b Confounder-adjusted rate difference and rate ratios are based on imputation analysis for missing covariates (shown in Table 1)

c Bias-corrected rate ratios refers to multiple probabilistic bias-corrected risk ratio, after simultaneous corrections for nondifferential exposure misclassification (infertility treatment) and unmeasured confounding biases

d A negative upper confidence limit for NNE means that the confidence interval contains two areas: lower limit to infinity and infinity to upper limit.

## Discussion

The timely discovery and focused management of GDM are of paramount importance because of their associations with several poor obstetric and neonatal outcomes (3, 20). In this large population-based study, we found that infertility treatment was associated with an 18%–30% increased risk of GDM across all BMIs as well as race and ethnicity groups. The GDM risk was more apparent with fertility-enhancing drugs compared with ART. These associations were stronger after corrections for exposure misclassification. The NNE across all categories highlights the public health significance and clinical relevance of the association between infertility treatment and GDM.

Providers involved in prenatal care must be attentive to their patients’ mode of conception, because this can change pregnancy management. For instance, developing GDM after pregnancies using ARTs may lead to worse perinatal outcomes than seen in women with GDM occurring after spontaneously conceived pregnancies (21). In addition, providers must be vigilant with GDM testing, because the rate of GDM is increasing worldwide because of the increase in maternal age and obesity, two well-known risk factors for GDM (2, 3, 22). Per the American College of Obstetricians and Gynecologists, those who are overweight or obese and who have additional risk factors, such as a history of GDM, a prior infant with a birth weight >4,000 g, a history of PCOS, or a high-risk racial and ethnic group, should be screened for GDM early in pregnancy (3).

A recent study using US vital statistics data (2011–2019) found that the rates of GDM had increased across all race and ethnicity groups in singleton first live births and were highest among non-Hispanic Asian, Hispanic, and Latina groups compared with the non-Hispanic White group (2). In addition, the rate of GDM was lower for non-Hispanic Black women in 2019 (55.7.7 per 1,000 live births) compared with non-Hispanic White women (57.7 per 1,000 live births) (RR 0.97, 95% CI 0.94–0.99) (2). Our study sheds further light on how infertility treatment influences the race-GDM relationship. For those with a normal BMI or above, the risk of GDM was similarly increased across all racial groups for any type of infertility treatment, and the risk was most pronounced for those using fertility-enhancing drugs, especially for Hispanic women. After corrections for biases, the association between infertility treatment and GDM risk was strongest for non-Hispanic Black women. However, our analysis did not stratify by additional races or ethnicities, such as the non-Hispanic Asian subgroup. Although infertility treatment seems to increase the risk of GDM across all racial subgroups, certain groups may have a greater propensity for the development of GDM, depending on the type of infertility treatment used and starting BMI.

Even after correction for confounders and unmeasured biases, infertility treatment appears to be an independent risk factor for GDM. This finding is in accordance with other studies (6, 7, 23, 24). In a meta-analysis of 38 studies (7), pregnancies using ARTs (n = 63,760) had a 53% higher risk of GDM compared with spontaneous conceptions (n = 1,800,000), and the risk was greater in those undergoing IVF (RR 1.95, 95% CI 1.56–2.44) and fresh embryo cycles (RR 1.38, 95% CI 1.03–1.85).

The risk of GDM was greater for those using fertility-enhancing drugs than for ARTs compared with spontaneous conceptions. Clomiphene citrate, letrozole, and exogenous gonadotropins are common first-line treatment options that are used either alone or in combination for ovulation induction, with or without IUI (25). The literature to date has not clearly defined differences in rates of GDM caused by fertility-enhancing drugs and IUI vs. IVF or ICSI. As a matter of fact, most studies either include both IVF and ICSI as well as IUI into one umbrella “ART” group or do not specify (6, 26). Ashrafi et al. (6) divided their analysis using IVF and ICSI as well as IUI and found a greater percentage of those in the IVF and ICSI group developing GDM (43.1%) vs. those in the IUI group (26%). Similarly, compared with controls, Maman et al. (27) found a higher rate of GDM in the IVF group (21.3% vs. 11.3%) and ovulation induction group (12.7% vs. 6.9%); however, the rate of GDM in the IVF group was not compared with the rate in the IUI group (27). These findings are in contrast to our findings that GDM risk is greater in the fertility-enhancing drugs group compared with the ART group. This finding is perplexing because oftentimes many patients who undergo ARTs have already attempted treatment with fertility-enhancing drugs; this finding certainly warrants further investigation.

With regard to prepregnancy BMI, current studies in the literature analyzing this relationship have mainly found greater risks of GDM in overweight or obese individuals (8, 9, 28). In a prospective cohort study, prepregnancy BMI ≥25 kg/m^2^ and inappropriate weight gain during pregnancy, specifically below recommended weight gain, were associated with increased risks of GDM in both ART-induced and spontaneously-induced conceptions (8). A nested case-control study (9) showed that, compared with those without GDM, patients receiving ART treatment (IVF or ICSI) with GDM were older, had a higher prepregnancy BMI, and had a history of PCOS. The combination of BMI ≥25.4 kg/m^2^ and first-trimester fasting blood glucose levels >84.5 mg/dL was predictive of GDM (sensitivity 70.7% and specificity 80.6%) (9). Additionally, a matched case-control study (29) of patients in the IVF-GDM group (cases) and non-IVF-GDM group (controls) found that first-trimester fasting glucose was slightly higher among cases than controls. These studies suggest that although prepregnancy BMI is a consideration, possibly early fasting blood glucose is more important than prepregnancy BMI alone.

Why are women who undergo infertility treatment at greater risk of developing GDM? Increased maternal age in the infertility population is certainly a consideration, although age was adjusted in all analyses in our study. Some have postulated that the higher GDM risk may be because of the underlying infertility etiology, the specific fertility medications, or the underlying mechanisms and laboratory techniques (30, 31, 32, 33). Gestational diabetes mellitus has been attributed largely to increased insulin resistance driven by various hormones, including estrogen, progesterone, and placenta-related proteins. In two separate studies, progesterone use in pregnancy had a significant association with GDM, possibly explained by progesterone’s known impact on insulin resistance (5, 6). Perhaps fertility medications are altering insulin sensitivity and glucose tolerance. On the other hand, possibly the laboratory mechanisms are altering glucocorticoid hormones and receptor levels. In mouse models, IVF and embryo culture have been shown to produce glucose intolerance (34). There is still much to be discovered regarding the underlying pathophysiology of infertility treatment and the development of GDM.

Polycystic ovary syndrome has been associated with GDM (35, 36), and many patients with PCOS undergo infertility treatment. However, in studies that controlled for PCOS, rates of GDM were still increased in the ART groups compared to the spontaneous pregnancy groups (6). How BMI modifies the PCOS-GDM relationship is still up for debate. In a case-control study (37), those with GDM had a higher rate of PCOS than those without GDM, but this relationship was not modified using BMI. Likewise, no differences in the rate of GDM were reported when patients with PCOS were matched by age and weight to the control group (38). In our study, although the RRs_bC_ may account for certain unmeasured confounders, we were unable to specifically account for PCOS because of data unavailability.

Another factor to consider is male partner obesity. In a large, retrospective cohort study, increased odds of infertility for overweight (odds ratio 1.20, 95% CI 1.04–1.38) and obese men (odds ratio 1.38, 95% CI 1.13–1.63) were reported (39). Although our study does not take into consideration male partner BMI, this is certainly an area for future exploration.

### Strengths of the Study

A major strength of this study is the use of a very large database that encompasses virtually all live births and stillbirths delivered at ≥20 weeks in the United States. This affords generalizable findings. Additionally, the analysis was corrected for unknown confounders and misclassification biases. Infertility treatment is likely underreported on vital records and certificates (16). As such, adjusting for misclassification bias was necessary for the analysis and has been shown previously to be a valid method for adjusting for such biases (40). The imputation methodology for missing covariate data minimizes selection bias (had we excluded patients with missing data) and may have resulted in a lack of generalizability of findings.

### Limitations of the Study

The reporting of data into two broad subgroups of infertility treatment does not allow us to examine outcomes by specific infertility treatment (i.e., IVF vs. IUI), and data on PCOS, a probable risk factor for GDM (35, 36, 41, 42), was unavailable. Compounding this is the fact that infertility treatment is not reported accurately on vital statistics forms (16) and mainly relies on information abstracted from either the prenatal care records or labor and delivery nursing triage notes. Additionally, certain racial subgroups may be unlikely to report the use of infertility treatment at the time of intake. Information on GDM may be subject to (differential) recall bias. There are other potential uncontrolled factors that may modify the infertility-GDM relationship. For example, our analysis was limited to prepregnancy BMI; we are not able to analyze outcomes by first- or second-trimester BMI. In addition, we could not differentiate between diet-controlled GDM and medication-controlled GDM.

## Conclusions

Infertility treatment should be considered an independent risk factor for GDM, and special attention should be given to those receiving fertility-enhancing drugs. Our study provides a framework for more targeted screening in this population. All infertility specialists must be vigilant with preconception counseling and ensure that all patients, regardless of race or prepregnancy BMI, are adequately tested for GDM early in pregnancy. Providers must recognize that certain racial subgroups may have a greater propensity for the development of GDM. As infertility specialists have the unique advantage of being in contact with the patient early on in pregnancy, we recommend testing as early as possible by a fasting blood glucose or by the usual conventional methods, which entails a 1-hour 50-g oral glucose tolerance test, by the infertility specialist. However, testing can be deferred once the patient comes in first contact with the prenatal care provider (i.e., general obstetrician or maternal-fetal medicine specialist), as patients are generally discharged from infertility practices around 8–10 weeks’ gestation and infertility specialists generally do not manage GDM during pregnancies. Consideration should be made to include pregnancies conceived via any infertility treatment type to The American College of Obstetricians and Gynecologists’ predefined list of criteria for earlier diabetes screening.

### CRediT Authorship Contribution Statement

**Devika Sachdev:** Conceptualization, Writing – original draft, Writing – review & editing. **Mark V. Sauer:** Conceptualization, Supervision, Writing – review & editing. **Cande V. Ananth:** Conceptualization, Data curation, Formal analysis, Investigation, Methodology, Project administration, Software, Supervision, Validation, Writing – review & editing.

## Declaration of Interests

D.S. has nothing to disclose. M.V.S. has nothing to disclose. C.V.A. has nothing to disclose.
