# Supplementary material for: Gestational diabetes mellitus in pregnancies conceived after infertility treatment: a population-based study in the United States, 2015–2020
Source: F S Rep. 2023 Nov 17;5(1):102–10. doi: 10.1016/j.xfre.2023.11.008 (PMC10958713; doi:10.1016/j.xfre.2023.11.008)
Supplement: Supplemental Table 1 [file mmc1.docx]

**Supplemental Table 1**

**Risk of gestational diabetes mellitus by assisted reproductive technology and stratified by race/ethnicity and BMI (kg/m^2^) among singleton births: United States, 2015-2020**

| **Assisted reproductive technology and pre-pregnancy body mass index (kg/m^2^)** | **Adjusted rate difference (95% CI)** | **Adjusted number needed to be exposed (95% CI)** | **Adjusted rate ratio (95% CI)^a,b^** |
| --- | --- | --- | --- |
|  |  |  |  |
| **Spontaneous conceptions** | 0.0 (Reference) | 0 (Reference) | 1.00 (Reference) |
|  |  |  |  |
| **Non-Hispanic White** |  |  |  |
| Underweight (<18.5) | 1.2 (0.4, 2.0) | 168 (84, -13591) | 1.25 (1.06, 1.48) |
| Normal weight (18.5-24.9) | 1.1 (0.9, 1.3) | 114 (97, 137) | 1.25 (1.21, 1.29) |
| Overweight (25-29.9) | 2.0 (1.6, 2.3) | 66 (55, 82) | 1.24 (1.20, 1.28) |
| Class I obesity (30-34.9) | 2.0 (1.4, 2.6) | 79 (56, 132) | 1.14 (1.09, 1.19) |
| Class II and III obesity (≥35) | 2.8 (2.2, 3.5) | 72 (48, 141) | 1.13 (1.09, 1.18) |
|  |  |  |  |
| **Non-Hispanic Black** |  |  |  |
| Underweight (<18.5) | 1.7 (-1.8, 5.3) | 274 (40, -56) | 0.87 (0.29, 2.64) |
| Normal weight (18.5-24.9) | 1.4 (0.6, 2.3) | 381 (121, -333) | 1.26 (1.10, 1.45) |
| Overweight (25-29.9) | 2.6 (1.5, 3.7) | 135 (66, -2842) | 1.26 (1.14, 1.40) |
| Class I obesity (30-34.9) | 2.8 (1.2, 4.5) | 82 (40, -2989) | 1.20 (1.07, 1.35) |
| Class II and III obesity (≥35) | 3.0 (0.8, 5.1) | 193 (47, -92) | 1.12 (0.99, 1.26) |
|  |  |  |  |
| **Hispanic** |  |  |  |
| Underweight (<18.5) | 1.9 (-1.4, 5.1) | 112 (36, -102) | 1.34 (0.81, 2.22) |
| Normal weight (18.5-24.9) | 0.5 (-0.2, 1.1) | 717 (169, -319) | 1.16 (1.06, 1.26) |
| Overweight (25-29.9) | 2.2 (1.2, 3.2) | 99 (56, 437) | 1.26 (1.17, 1.35) |
| Class Iobesity (30-34.9) | 2.1 (0.5, 3.7) | 57 (33, 220) | 1.18 (1.08, 1.28) |
| Class II and III obesity (≥35) | 1.9 (-0.3, 4.1) | 2952 (56, -58) | 1.05 (0.95, 1.16) |
|  |  |  |  |

CI, Confidence Interval; SI, simulation interval

^a^ Rate ratios are adjusted for maternal age, live-born parity, education, race/ethnicity, BMI, chronic hypertension, year of delivery

^b^ Confounder-adjusted rate difference and rate ratios are based on imputation analysis for missing covariates (shown in Table 1)

^c^ Bias corrected RRs refers to multiple probabilistic bias-corrected risk ratio, following simultaneous corrections for non-differential exposure misclassification (infertility treatment) and unmeasured confounding biases

^d^ A negative upper confidence limit for NNE means that the confidence interval contains 2 areas: lower limit to infinity and -infinity to upper limit
