# Supplementary material for: Gestational diabetes mellitus in pregnancies conceived after infertility treatment: a population-based study in the United States, 2015–2020
Source: F S Rep. 2023 Nov 17;5(1):102–10. doi: 10.1016/j.xfre.2023.11.008 (PMC10958713; doi:10.1016/j.xfre.2023.11.008)
Supplement: Supplemental Table 2 [file mmc2.docx]

**Supplemental Table 2**

**Risk of gestational diabetes mellitus by Fertility-Enhancing Drugs and stratified by race/ethnicity and BMI (kg/m^2^)
among singleton births: United States, 2015-2020**

| **Fertility-enhancing drugs and pre-pregnancy body mass index (kg/m^2^)** | **Adjusted rate difference (95% CI)** | **Number needed to be exposed (95% CI)** | **Adjusted rate ratio (95% CI)^a,b^** |
| --- | --- | --- | --- |
|  |  |  |  |
| **Spontaneous conceptions** | 0.0 (Reference) | 0 (Reference) | 1.00 (Reference) |
|  |  |  |  |
| **Non-Hispanic White** |  |  |  |
| Underweight (<18.5) | 1.0 (0.1, 1.9) | 145 (68, -1065) | 1.27 (1.03, 1.56) |
| Normal weight (18.5-24.9) | 1.0 (0.8, 1.3) | 116 (94, 150) | 1.25 (1.20, 1.31) |
| Overweight (25-29.9) | 2.4 (2.1, 2.8) | 45 (38, 54) | 1.32 (1.27, 1.38) |
| Class I obesity (30-34.9) | 3.6 (2.3, 4.2) | 31 (26, 38) | 1.28 (1.23, 1.33) |
| Class II and III obesity (≥35) | 5.3 (4.7, 5.9) | 22 (19, 26) | 1.29 (1.24, 1.34) |
|  |  |  |  |
| **Non-Hispanic Black** |  |  |  |
| Underweight (<18.5) | 0.9 (-3.2, 4.9) | 82 (20, -37) | 1.41 (0.50, 4.02) |
| Normal weight (18.5-24.9) | 1.2 (0.1, 2.2) | 288 (87, -218) | 1.21 (1.00, 1.48) |
| Overweight (25-29.9) | 3.2 (1.7, 4.7) | 61 (36, 212) | 1.33 (1.15, 1.53) |
| Class I obesity (30-34.9) | 3.2 (1.3, 5.2) | 59 (30, 4974) | 1.18 (1.02, 1.37) |
| Class II and III obesity (≥35) | 6.3 (3.8, 8.8) | 20 (14, 35) | 1.41 (1.24, 1.60) |
|  |  |  |  |
| **Hispanic** |  |  |  |
| Underweight (<18.5) | 1.8 (-2.0, 5.5) | 95 (26, -57) | 1.47 (0.74, 2.94) |
| Normal weight (18.5-24.9) | 1.5 (0.7, 2.2) | 86 (55, 202) | 1.33 (1.19, 1.47) |
| Overweight (25-29.9) | 3.4 (2.2, 4.5) | 41 (29, 73) | 1.38 (1.26, 1.52) |
| Class I obesity (30-34.9) | 5.6 (3.9, 7.4) | 19 (14, 27) | 1.42 (1.29, 1.55) |
| Class II and III obesity(≥35) | 4.6 (2.6, 6.6) | 27 (18, 55) | 1.23 (1.12, 1.35) |
|  |  |  |  |

CI, Confidence Interval; SI, simulation interval

^a^ Rate ratios are adjusted for maternal age, live-born parity, education, race/ethnicity, BMI, chronic hypertension, year of delivery

^b^ Confounder-adjusted rate difference and rate ratios are based on imputation analysis for missing covariates (shown in Table 1)

^c^ Bias corrected RRs refers to multiple probabilistic bias-corrected risk ratio, following simultaneous corrections for non-differential exposure misclassification (infertility treatment) and unmeasured confounding biases

^d^ A negative upper confidence limit for NNE means that the confidence interval contains 2 areas: lower limit to infinity and -infinity to upper limit
