# Supplementary material for: Gestational diabetes mellitus in pregnancies conceived after infertility treatment: a population-based study in the United States, 2015–2020
Source: F S Rep. 2023 Nov 17;5(1):102–10. doi: 10.1016/j.xfre.2023.11.008 (PMC10958713; doi:10.1016/j.xfre.2023.11.008)
Supplement: Supplemental Figures [file mmc3.docx]

**Supplemental Figure 1**

Total US singleton births, 2015 – 2020
 (n = 23,274,997)

Twins and higher-order pregnancies (n = 797,594)

Pregestational diabetes diagnosis (n = 363,251)

Delivery at <20 weeks or ≥45 weeks (n = 140,212)

Missing gestational age (n = 17,556)

Total Exclusions (n = 1,332,707)

Singleton births between 20 – 44 weeks’ gestation
(n = 21,943,384)
